# Targeted Genetic Sequencing Analysis of 223 Cases of Pseudomyxoma Peritonei Treated by Cytoreductive Surgery and Hyperthermic Intraperitoneal Chemotherapy Shows Survival Related to *GNAS* and *KRAS* Status

**DOI:** 10.1002/cam4.70340

**Published:** 2024-10-22

**Authors:** Jane Gibson, Reuben J. Pengelly, Amatta Mirandari, Konstantinos Boukas, Sophia Stanford, Thomas Desmond Cecil, Faheez Mohamed, Sanjeev Paul Dayal, Alexios Tzivanakis, Brendan John Moran, Alex Mirnezami, Norman John Carr, Sarah Ennis

**Affiliations:** ^1^ Cancer Sciences, Faculty of Medicine University of Southampton Southampton UK; ^2^ Bio‐R Bioinformatics Research Facility, Faculty of Medicine University of Southampton Southampton UK; ^3^ Human Development and Health, Faculty of Medicine University of Southampton Southampton UK; ^4^ Wessex Investigational Sciences Hub, Cancer Sciences, Faculty of Medicine University of Southampton, Southampton General Hospital Southampton UK; ^5^ Peritoneal Malignancy Institute Basingstoke and North Hampshire Hospital Basingstoke UK

## Abstract

**Background and Aim:**

Pseudomyxoma peritonei (PMP) is an unusual condition with unique behaviour caused by a mucinous neoplasm, usually arising from the appendix. The aim of this study was to evaluate the prevalence of genomic alterations in clinical specimens of PMP using a targeted assay and correlate the findings with clinical, pathological and outcome data. Sequencing data from 223 patients were analysed.

**Results:**

The median follow‐up interval was 48 months. The primary neoplasm was appendiceal in 216 patients, ovarian in 4, urachal in 2 and renal in one. We confirmed common mutations in *GNAS* and *KRAS* (42% each) with significant co‐occurrence of variants in these genes. *TP53* mutations were found in 8%. Other mutations were rare but included novel mutations in *BAP1* and *ERBB4*. Of 17 patients with acellular peritoneal mucin, 6 (35%) were positive for DNA mutations. The non‐appendiceal cases generally showed a similar mutational landscape to the appendiceal lesions with *GNAS* and *KRAS* commonly mutated, although one urachal lesion showed multi‐hit *TP53* mutation without variants in either *GNAS* or *KRAS*. Survival was significantly associated with the grade of the primary neoplasm, the grade of the peritoneal disease, the completeness of cytoreduction score and with mutation in either *GNAS*, *KRAS* or both. The hazard ratio (HR) associated with mutation in *GNAS* and/or *KRAS* was 1.87 (*p* = 0.004).

**Conclusions:**

Survival outcome was more closely associated with the grade of the peritoneal disease than with the grade of the primary neoplasm. Our findings support the developing concept that mutational analysis may provide prognostic information in patients with PMP.

**Abbreviations**
AM: acellular mucin
CC: completeness of cytoreduction
G: grade
HAMN: high‐grade appendiceal neoplasm
HG: high grade
HGSC: high‐grade with signet‐ring cells
HR: hazard ratio
LAMN: low‐grade appendiceal neoplasm
LG: low grade
MAC: mucinous adenocarcinoma
NGS: next‐generation sequencing
PMP: pseudomyxoma peritonei
PSOGI: Peritoneal Surface Oncology Group International
SRC: mucinous adenocarcinoma with signet‐ring cells
VAF: variant allele frequency
WHO: World Health Organisation

## 1 Introduction

Pseudomyxoma peritonei (PMP) is a syndrome characterised by the accumulation of mucinous ascites and tumours within the abdominopelvic cavity [1, 2]. The incidence of PMP is about 2–3 per million per year [3, 4]. Typical features include abundant mucinous ascites, peritoneal implants of mucinous material, thickening of the omentum (‘omental cake’) and Krukenberg tumours of the ovaries. PMP has unusual features not shared with other types of neoplasm. In particular, it spreads widely through the peritoneal cavity by following the physiological flow of peritoneal fluid, a process that has been named the redistribution phenomenon, and it tends to push its way into underlying organs rather than frank tumour infiltration. Furthermore, at least in its low‐grade form, PMP very rarely metastasises to lymph nodes or distant organs [5, 6]. There is a spectrum of diseases varying from indolent low‐grade to more aggressive variants that are more invasive. Nevertheless, PMP is a malignant condition in all grades: if untreated, it grows relentlessly and ultimately causes death, usually through intestinal obstruction. For this reason, many authors prefer the term ‘mucinous carcinoma peritonei’ to PMP [7, 8].

By far the most common cause of PMP is a primary mucinous neoplasm of the appendix. There are four main types of mucinous appendiceal neoplasm according to the Peritoneal Surface Oncology Group International (PSOGI) classification: low‐grade appendiceal mucinous neoplasm (LAMN), high‐grade appendiceal mucinous neoplasm (HAMN), mucinous adenocarcinoma and mucinous adenocarcinoma with signet ring cells [7]. When one of these tumours ruptures, neoplastic cells are released into the peritoneal cavity where they can continue to grow and produce PMP. On rare occasions, PMP can develop from other primary tumours, for example, mucinous tumours of the urachus, ovarian teratomas, intraductal papillary mucinous neoplasms of the pancreas and mucinous neoplasms arising in retrorectal hamartomas [2, 9, 10].

The histological grade (G) of PMP is an independent predictor of prognosis [11]. There are three grades of PMP in the World Health Organization (WHO) classification: G1 is low grade, G2 is high grade, and G3 is high grade with signet ring cells [12, 13]. Tumours with signet‐ring cells are designated separately because of their worse prognosis [14]. In PMP of appendiceal origin, the grades of the appendiceal primary and the peritoneal disease are usually the same, but in 4%–5% of patients, there is discordance in grade. In these cases, it has been found that the grade of the PMP is more closely associated with prognosis, although the evidence is relatively scanty [15].

In about 10%–15% of patients with PMP syndrome, the intra‐abdominal mucin contains no neoplastic cells when it is examined histologically [16, 17]. This acellular mucin is not graded in the WHO classification. In the majority of such cases, the disease does not progress [18].

Appendiceal neoplasia and PMP are genetically distinct from colorectal carcinoma [19, 20]. Studies have shown the most frequent mutations in PMP to be in *KRAS* and *GNAS*, with frequencies of about 70% and 50%, respectively [21, 22, 23]. Most *KRAS* mutations are found in codon 12 (G12D, G12C and G12V) and in codon 13 (G13D) [24]. *TP53* is mutated in some cases, but this finding is usually confined to high‐grade PMP [25, 26]. Other genes exhibiting mutation in PMP include *FAT3/4*, *TGFBR1/2*, *RNF43*, *PIK3CA*, *CTNNB1*, *AKT1*, *ATM*, *SMAD4*, *SMAD2*, *ARID1A/B*, *ARID2*, *RBM10*, *BRCA2*, *MML*, *MLL2/3* and *CDKN2A* [21, 24, 27, 28, 29, 30]. *BRAF* mutations occur occasionally but are uncommon [31]. Studies using whole‐exome sequencing also identified mutations in *SMAD3*, *PRKACA*, *PRKAR1A*, *TCHH*, *HERC2*, *SPEG*, *TGFBR2*, *TTN*, *MUC17*, *PMEPA1*, *AHNAK*, *AHNAK2*, *APOB*, *FCGPB*, *HRNR* and *OBSCN* [20, 32]. One study demonstrated amplification of *MYC* in a subset of patients [30]. In contrast to colorectal carcinoma, it is unusual to find either *APC* mutations or microsatellite instability in PMP [22, 28, 31, 33]. Furthermore, on the rare occasions that microsatellite instability is found in appendiceal neoplasia, it is not generally associated with *MLH1* hypermethylation [34].

A study of appendiceal adenocarcinomas found that neoplasms that were *KRAS* mutant and *GNAS* wild type were associated with better overall survival than neoplasms with mutations in both genes [19], and a study of PMP of appendiceal origin found a mutation in any one of *TP53*, *SMAD4*, *ATM*, *CDKN2A*, *PIK3CA* or *PTEN* was associated with worse overall survival [28]. However, in general, there is little consensus in the literature concerning the prognostic implications of the various genetic mutations, partly because many studies include relatively few patients [21, 24].

The optimal treatment of PMP includes complete tumour removal by radical cytoreductive surgery combined with hyperthermic intraperitoneal chemotherapy, with overall 10‐year survival of over 50% [35, 36]. Occasional patients with non‐resectable recurrent disease may undergo intestinal transplantation [37]. Indicators of likely clinical outcome include the extent of intraperitoneal disease, the histological grade, preoperative serum concentrations of the tumour markers CA125, CA19‐9 and CEA and completeness of tumour removal [38, 39]. However, these clinical and pathological parameters do not accurately predict outcomes in all cases, and a better knowledge of genetic changes in PMP will contribute to the search for improved biomarkers of prognosis [32]. Furthermore, because of the unique behaviour of PMP, an increased understanding of its genetic landscape could shed light on mechanisms of metastasis in general and have implications beyond the treatment of this rare condition [40].

The aim of this study was to apply next‐generation sequencing (NGS) techniques to clinical specimens of pseudomyxoma peritonei by evaluating the prevalence of genomic alterations with a targeted NGS assay and to correlate the results with clinical, pathological and outcome data. A secondary aim was to compare the survival outcomes in patients with PMP of appendiceal origin according to the grade of the primary tumour compared with the grade of the peritoneal disease.

## 2 Materials and Methods

### 2.1 Patient Sample Collection/Criteria

Patients treated at the Peritoneal Malignancy Institute, Basingstoke, UK were recruited to the study. All patients provided written informed consent. All patients had pseudomyxoma peritonei treated by cytoreductive surgery and hyperthermic intraperitoneal chemotherapy. Ethical approval was provided by the National Research Ethics Service, reference 09/H0504/3.

Samples of tumour were taken intra‐operatively during surgery; these samples were snap‐frozen and stored in liquid nitrogen until further processing. Histological examination of all specimens was performed by a pathologist with a special interest in the appendix and PMP. The appendiceal primary tumours were classified according to PSOGI criteria as follows (WHO grade in parentheses): LAMN (G1), HAMN (G2), mucinous adenocarcinoma (G2) and mucinous adenocarcinoma with signet ring cells (G3). The peritoneal disease was classified as acellular mucin (ungraded), low‐grade PMP (G1), high‐grade PMP (G2) and high‐grade PMP with signet ring cells (G3) [7, 41].

Clinical details were retrieved from a prospectively maintained database supplemented by reference to the clinical records if required. The presence of any residual disease at the end of the operation was recorded by the surgeon using the completeness of cytoreduction (CC) score: no visible disease (CC0), nodules of residual tumour less than 0.25 cm diameter (CC1), nodules 0.25–2.5 cm diameter (CC2) and nodules more than 2.5 cm diameter (CC3). For modelling, the CC score was dichotomised into CC0/1 and CC2/3, corresponding to a cutoff value of 0.25 cm diameter, consistent with findings that this is the most clinically significant distinction [35, 42].

### 2.2 DNA Extraction, Panel Design, Library Preparation and Sequencing

DNA was extracted from fresh frozen samples. Firstly, samples were homogenised using a Precellys 24 in 2 mL CKMix tissue homogenising tubes (Bertin Technologies SAS) at 5000 rpm for two 10 s periods. Following homogenisation, DNA was extracted using the QIAamp DNA Blood Mini kit, automated using the QIASymphony platform (Qiagen). DNA samples were quantified using the Qubit Fluorimeter (Life Technologies).

A custom‐targeted panel was designed for peritoneal malignancies, including those underlying PMP. This panel included 207 key regions across 50 genes frequently mutated across cancers, and the coding regions of six key genes of interest in peritoneal malignancy (*BAP1*, *CDC42*, *NF2*, *RNF43*, *SETDB1* and *TRAF7*) (Table S1). In addition, 24 SNPs were included to allow verification of sample identity following sequencing [43]. The custom panel was generated using an amplicon‐based approach (YouSeq, Winchester, UK). Capture and library preparation was performed in accordance with manufacturer's instructions. Libraries were paired‐end sequenced (300 cycles) on a NextSeq500. Parallel genotyping of the 24‐sample tracking panel was performed using KASP genotyping (LGC Genomics, UK).

### 2.3 Bioinformatic and Statistical Analyses

Raw‐sequencing fastq files from 2 runs were merged per sample. Alignment of the merged sequences to the hg38 (release 13) genome was performed using the BWA‐MEM module from the Burrows‐Wheeler Aligner (BWA) software (v.0.7.17) [44] and sorted and indexed using samtools (v.1.16.1). The sorted bams were trimmed using the bamutil (V1.0.14) filter programme to trim ends of reads where there were > 10% mismatches from the reference genome and exclude reads where there were mismatches with a cumulative phred scaled quality of 60.

The trimmed bams were input into Stitcher (v5.2.9) to merge read pairs followed by variant calling and variant quality score recalibration using Pisces (v5.2.9). These variants were then annotated by the Functotator software (v.4.2.2) using base data sources and output as maf format files. These files were analysed in maftools 2.18.0, in Rstudio (R v4.3.2). Variants were filtered for quality score > 100 and variant allele frequency (VAF) ≥ 2.5% (1% for *GNAS/KRAS*). To exclude known germline variants, all variants were filtered to remove those present in gnomAD at a minor allele frequency of > 0.0001. Manual curation of all mutated genes was carried out using IGV (v. 2.14.1) and mutations were assessed and tagged for exclusion based on a published standard operating procedure [45].

Survival analyses were carried out using the ‘survival’ (3.5‐7), ‘survminer’ (0.4.9) and ‘forestmodel’ (0.6.2) R packages in Rstudio (R v4.3.2).

## 3 Results

### 3.1 Clinical Characteristics

Samples from 263 patients were submitted for analysis. In 223 samples, DNA was successfully extracted and good‐quality sequencing data were achieved (Table S2). The demographic and clinical details of these 223 patients are shown in Table 1. Pre‐operative chemotherapy had been received by 36 (16.1%) patients; the others had received no chemotherapy prior to cytoreductive surgery. Follow‐up was available for all patients and the median follow‐up interval was 50 months.

**TABLE 1:** Patient/sample demographic and clinical data according to the grade of the peritoneal disease.

	AM	LG (G1)	HG (G2)	HGSC (G3)	Overall
	(*N* = 17)	(*N* = 150)	(*N* = 50)	(*N* = 6)	(*N* = 223)
*Age at surgery*					
Mean (SD)	61.4 (11.1)	57.6 (12.0)	55.6 (13.3)	50.0 (17.0)	57.2 (12.5)
Median [Min, Max]	60.0 [43.0, 76.0]	58.0 [30.0, 85.0]	55.0 [27.0, 84.0]	49.0 [32.0, 78.0]	57.0 [27.0, 85.0]
*Sex*					
Female	12 (70.6%)	104 (69.3%)	29 (58.0%)	2 (33.3%)	147 (65.9%)
Male	5 (29.4%)	46 (30.7%)	21 (42.0%)	4 (66.7%)	76 (34.1%)
*Time from first surgery to follow up* (*months*)					
Mean (SD)	68.5 (35.9)	61.9 (33.8)	46.7 (34.0)	27.0 (21.1)	58.1 (34.6)
Median [Min, Max]	53.0 [19.0, 121]	57.5 [4.00, 145]	40.0 [4.00, 123]	26.0 [2.00, 61.0]	50.0 [2.00, 145]
*CC score*					
CC0 (no residual disease)	9 (52.9%)	33 (22.0%)	24 (48.0%)	2 (33.3%)	68 (30.5%)
CC1 (< 0.25 cm)	8 (47.1%)	76 (50.7%)	21 (42.0%)	2 (33.3%)	107 (48.0%)
CC2 (0.25–2.5 cm)	0 (0%)	8 (5.3%)	1 (2.0%)	0 (0%)	9 (4.0%)
CC3 (> 2.5 cm)	0 (0%)	28 (18.7%)	4 (8.0%)	2 (33.3%)	34 (15.2%)
Missing	0 (0%)	5 (3.3%)	0 (0%)	0 (0%)	5 (2.2%)
*Primary site*					
Appendix	16 (94.1%)	147 (98.0%)	47 (94.0%)	6 (100%)	216 (96.9%)
Ovarian teratoma	1 (5.9%)	2 (1.3%)	1 (2.0%)	0 (0%)	4 (1.8%)
Urachus	0 (0%)	1 (0.7%)	1 (2.0%)	0 (0%)	2 (0.9%)
Kidney	0 (0%)	0 (0%)	1 (2.0%)	0 (0%)	1 (0.4%)
*Primary tumour*					
LAMN	14 (82.4%)	145 (96.7%)	3 (6.0%)	0 (0%)	162 (72.6%)
HAMN	0 (0%)	0 (0%)	6 (12.0%)	0 (0%)	6 (2.7%)
Appendiceal MAC	2 (11.8%)	2 (1.3%)	36 (72.0%)	0 (0%)	40 (17.9%)
Appendiceal SRC	0 (0%)	0 (0%)	2 (4.0%)	6 (100%)	8 (3.6%)
PMP non‐appendiceal	1 (5.9%)	3 (2.0%)	3 (6.0%)	0 (0%)	7 (3.1%)
*Pre‐op CA19.9* (*U/mL*)					
Mean (SD)	48.6 (66.8)	862 (2570)	376 (965)	266 (378)	679 (2190)
Median [Min, Max]	16.0 [6.00, 251]	187 [0, 20,400]	121 [0, 6040]	48.0 [3.00, 879]	127 [0, 20,400]
Missing	1 (5.9%)	5 (3.3%)	4 (8.0%)	0 (0%)	10 (4.5%)
*Pre‐op CA125* (*U/mL*)					
Mean (SD)	25.1 (20.3)	58.2 (61.3)	75.2 (181)	74.3 (36.9)	59.9 (100)
Median [Min, Max]	18.0 [3.00, 73.0]	38.0 [2.00, 403]	25.3 [4.00, 1260]	75.5 [15.0, 122]	34.5 [2.00, 1260]
Missing	0 (0%)	6 (4.0%)	1 (2.0%)	0 (0%)	7 (3.1%)
*Pre‐op CEA* (*ng/mL*)					
Mean (SD)	25.2 (66.1)	61.9 (154)	50.2 (151)	13.6 (18.6)	55.0 (147)
Median [Min, Max]	4.70 [0.500, 272]	21.0 [0.500, 1650]	9.70 [0.500, 890]	6.85 [2.30, 51.0]	15.0 [0.500, 1650]
Missing	0 (0%)	6 (4.0%)	1 (2.0%)	0 (0%)	7 (3.1%)

Abbreviations: AM, acellular mucin; HAMN, high‐grade appendiceal neoplasm; HG, high grade; HGSC, high‐grade with signet ring cells; LAMN, low‐grade appendiceal neoplasm; LG, low grade; MAC, mucinous adenocarcinoma; SRC, signet ring cells.

The PMP originated from a mucinous neoplasm of the appendix in 216 (96.9%) patients (Table 2). The other primary tumours were an ovarian teratoma in 4 patients, a mucinous tumour of the urachus in 2 patients and a mucinous tumour of the renal pelvis in one patient. There were 17 (7.6%) patients with histologically acellular mucin; in 14 of them, the primary was an LAMN, in 2, the primary was an appendiceal mucinous adenocarcinoma and in one the primary was an ovarian teratoma. The presence or absence of lymph node metastasis was recorded in 166 (74.4%) patients. Nodal metastasis was present in 18/166 (10.8%) patients; 5 with G1 PMP, 11 with G2 PMP and 2 with G3 PMP. There was discordance between the grade of the appendiceal primary and the grade of the PMP in 7 (3.1%) patients: three G1 primaries were associated with G2 PMP, two G2 primaries were associated with G1 PMP, and two G3 primaries were associated with G2 PMP (Table 2).

**TABLE 2:** Grade of pseudomyxoma peritonei according to type of primary neoplasm.

		Peritoneal disease			
		AM	LG (G1)	HG (G2)	HGSC (G3)
Primary tumour	LAMN (G1)	14	145	3	0
	HAMN (G2)	0	0	6	0
	Appendiceal MAC (G2)	2	2	36	0
	Appendiceal SRC (G3)	0	0	2	6
	Non‐appendiceal primary	1	3	3	0

Abbreviations: AM, acellular mucin; HAMN, high‐grade appendiceal neoplasm; HG, high grade; HGSC, high grade with signet ring cells; LAMN, low‐grade appendiceal neoplasm; LG, low grade; MAC, mucinous adenocarcinoma; SRC, signet ring cells.

### 3.2 Mutational Landscape

**FIGURE 1:**
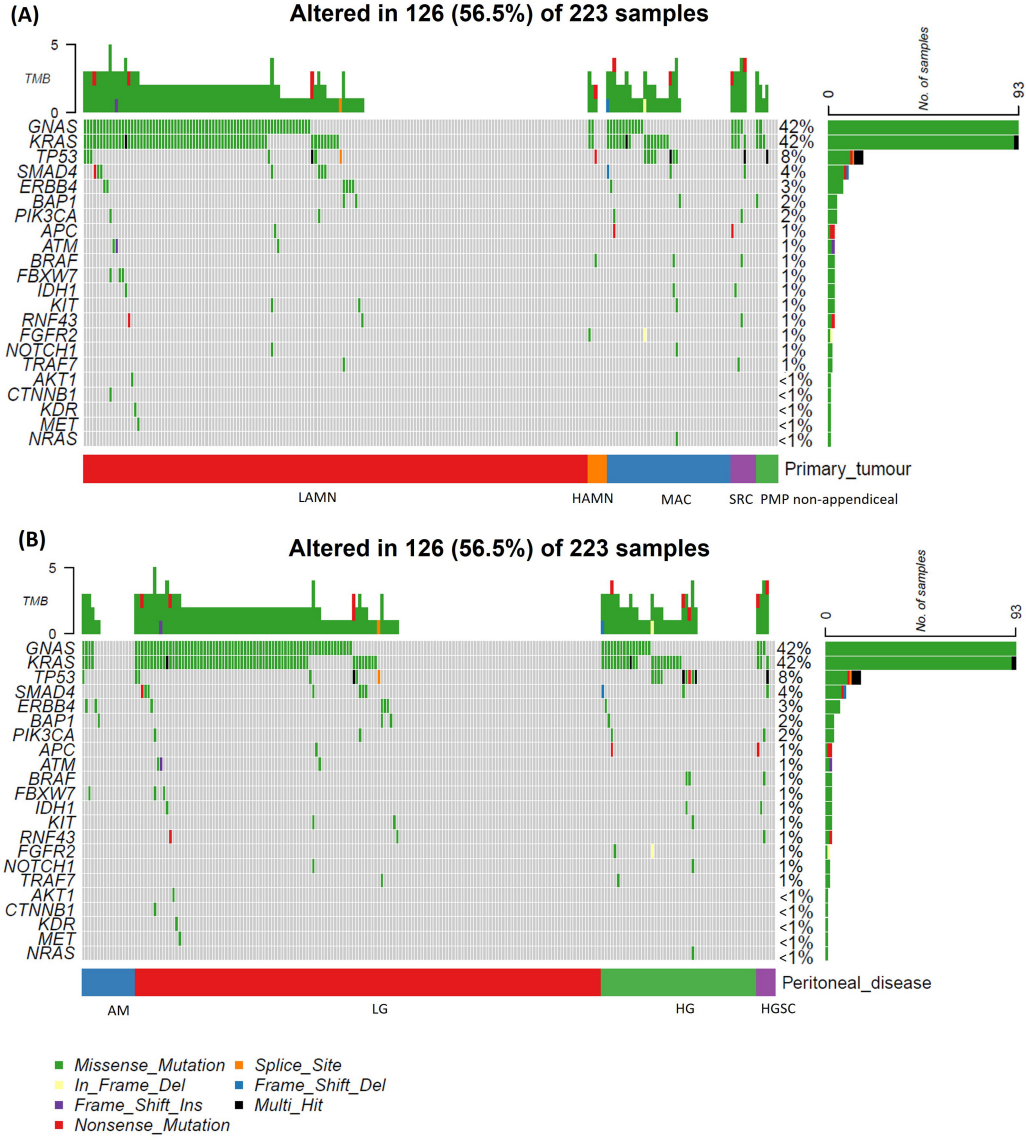
Oncoplot of mutations grouped by (A) primary tumour and (B) by peritoneal disease.

Of the 223 patients successfully sequenced across cancer hotspot regions, we observed good‐quality variants in 126 (56.5%) patients. The most commonly mutated genes were *GNAS* and *KRAS* at 42% each (Figure 1). Mutations in *KRAS* were most commonly seen at the known mutation hotspots in codons 12 and 13, and those in *GNAS* were at the known hotspot in codon 201 (Figure S2). *TP53* mutations were found in 8% and were more common in high‐grade lesions. *SMAD4*, *ERBB4*, *BAP1* and *PIK3CA* were detected at rates of 2%–4%. Mutations in *APC* and *BRAF* were unusual (1% each). Mutated *NRAS* was found in only one (0.4%) specimen, a mucinous appendiceal adenocarcinoma. In the 17 specimens consisting of acellular peritoneal mucin, 6 (35%) were positive for mutated DNA (Figure 1B).

The 7 lesions of non‐appendiceal origin generally showed a similar mutational landscape to the appendiceal lesions, with *GNAS* and *KRAS* variants the most frequent, although one patient had no mutation in either gene but did harbour multihit mutation of *TP53*; this lesion was a mucinous adenocarcinoma of the urachus (Figure 1A). One case of appendiceal mucinous adenocarcinoma also contained multihit mutation of *TP53* without mutation in either *KRAS* or *GNAS*.

### 3.3 Mutual Exclusivity Analysis

**FIGURE 2:**
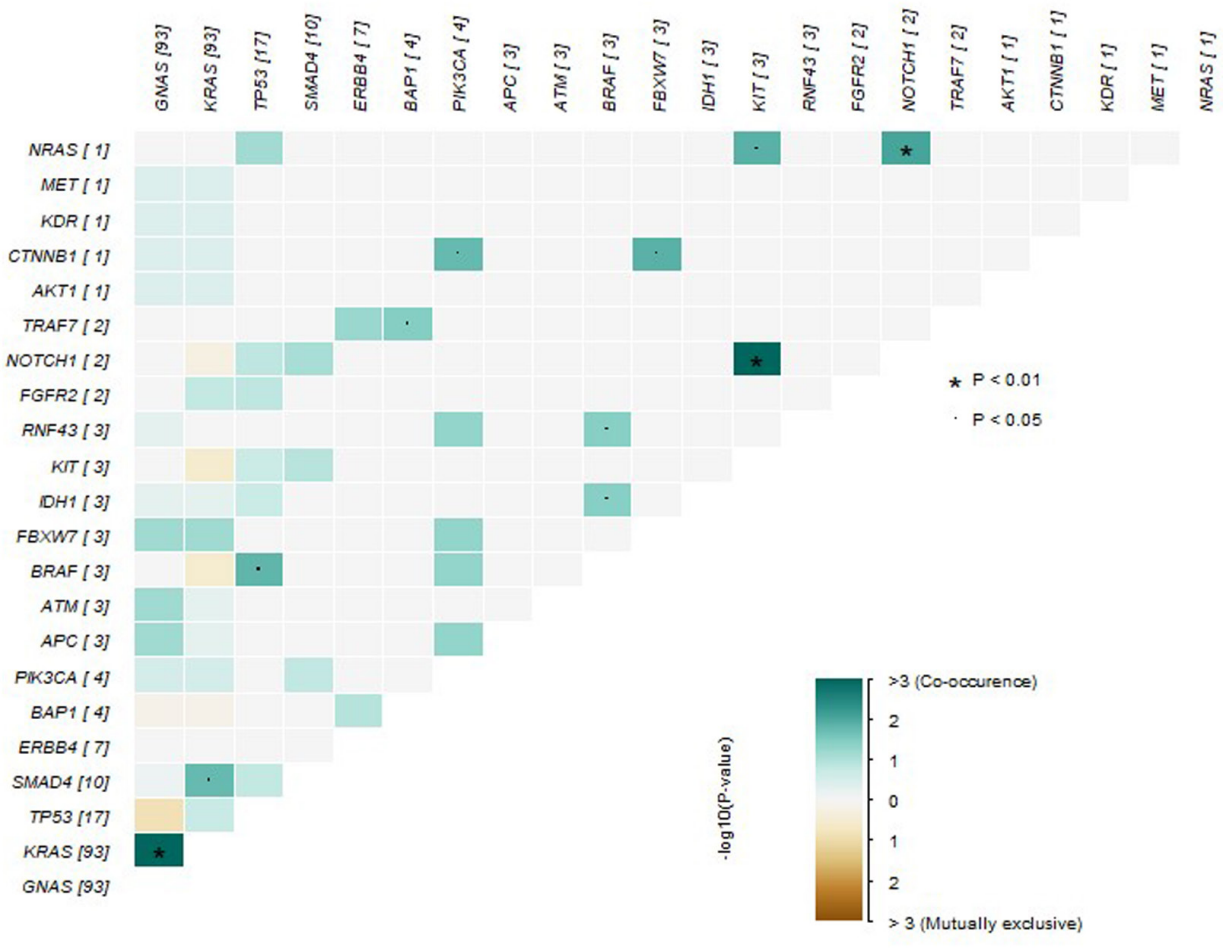
Mutual exclusivity and co‐occurrence of mutations.

Mutations in *GNAS* and *KRAS* significantly co‐occur (adj. *p*‐value 2.72 × 10^−21^, odds ratio 22.3). The most mutually exclusive mutations were between *GNAS* and *TP53* although this was not significant (adj. *p*‐value 0.9, odds ratio 0.4) (Figure 2). Mutations in *NOTCH1* and *KIT* were also significantly mutually inclusive (adj. *p*‐value 0.003), but this was based on very few mutations, where two patients with *NOTCH1* mutations both also had mutations in *KIT*.

### 3.4 Survival Analyses

The median survival for all patients with PMP of appendiceal origin was 96 months. Overall median survival for the non‐appendiceal group was 51% at 61 months (median survival not reached in this cohort). Modelling survival of patients using key clinical data identified CC score as the factor with most impact. The CC score was available for 218 patients; the median survival of the 175 patients with CC0/1 was 116 months and the median survival of the 43 patients with CC2/3 was 26 months (*p* = 2.57 × 10^−20^).

**FIGURE 3:**
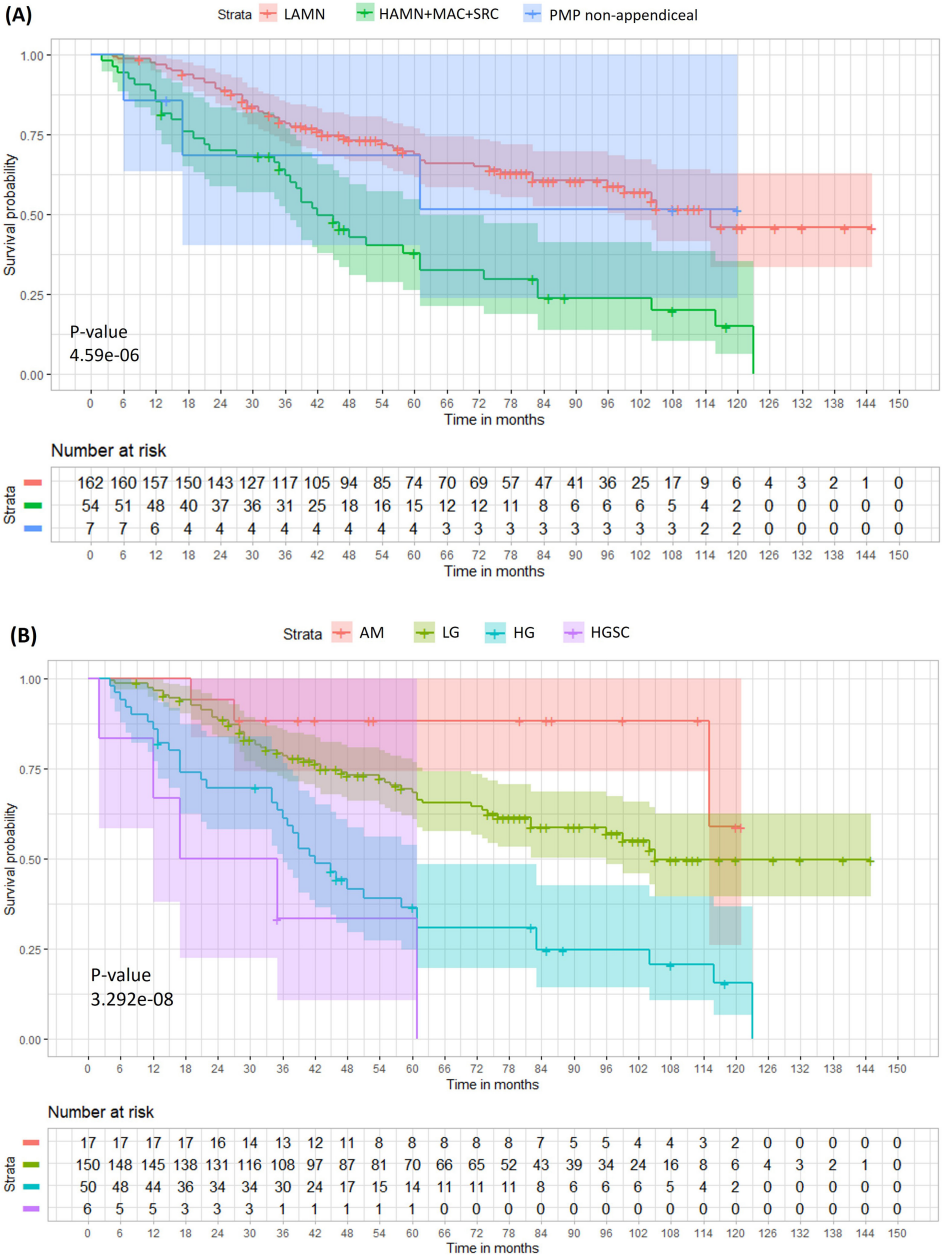
Kaplan–Meier Survival plot, stratified by (A) primary tumour and (B) peritoneal disease.

For the appendiceal neoplasms, survival rates differed significantly according to the grade of the primary tumour (Figure 3A). Dichotomising the primary tumours into LAMN (G1) and HAMN/mucinous adenocarcinoma/mucinous adenocarcinoma with signet ring cells (HAMN/MAC/SRC) (G2/3) showed the median overall survival for the LAMN group was 115 months and for the HAMN/MAC/SRC group 42 months (*p* = 4.59 × 10^−6^, HR = 4.06). The 5‐year survival was 69% and 38% for the LAMN and HAMN/MAC/SRC groups, respectively. The 5‐year survival for the non‐appendiceal group was 69%. Survival was also significantly different between peritoneal disease types (*p* = 3.292 × 10^−8^) (Figure 3B). The median survival for low‐grade PMP (G1) was 105 months, for high‐grade PMP (G2) 42 months and for high‐grade PMP with signet ring cells (G3) 26 months. In the 17 patients with acellular mucin, only three experienced recurrence and the 5‐year survival rate was 88%, compared with an overall 5‐year survival of 59% in the 206 patients with neoplastic cells in the peritoneal mucin (*p* = 0.035).

We compared survival outcomes of patients with PMP of appendiceal origin when classified according to the WHO grade of the appendiceal primary and according to the WHO grade of the PMP. Patients with acellular peritoneal mucin were excluded. The HR for primary tumour grade was 2.82 (*p* = 9.96 × 10^−9^) while the HR for peritoneal disease grade was higher, 3.82 (*p* = 2.91 × 10^−9^). In the Cox model with both variables included, PMP grade was more closely associated with survival and the primary tumour grade made no further contribution (*p* = 0.0002, Figure S5). These findings show that prognosis is more closely associated with the grade of the PMP than the grade of the appendiceal primary.

Comparing survival rates in patients with and without *KRAS* and *GNAS* mutations, individuals harbouring a *KRAS* variant had poorer survival than those without (*p* = 0.006, HR = 1.72). Similarly, those carrying a *GNA*S variant had poorer survival than those without (*p* = 0.048, HR = 1.48). The next most frequently mutated gene was *TP53* (mutated in 17); although there was a trend towards poorer survival in the mutated cases this was not significant (*p* = 0.061). There was no significant association between CC score and genetic mutation in our data.

**FIGURE 4:**
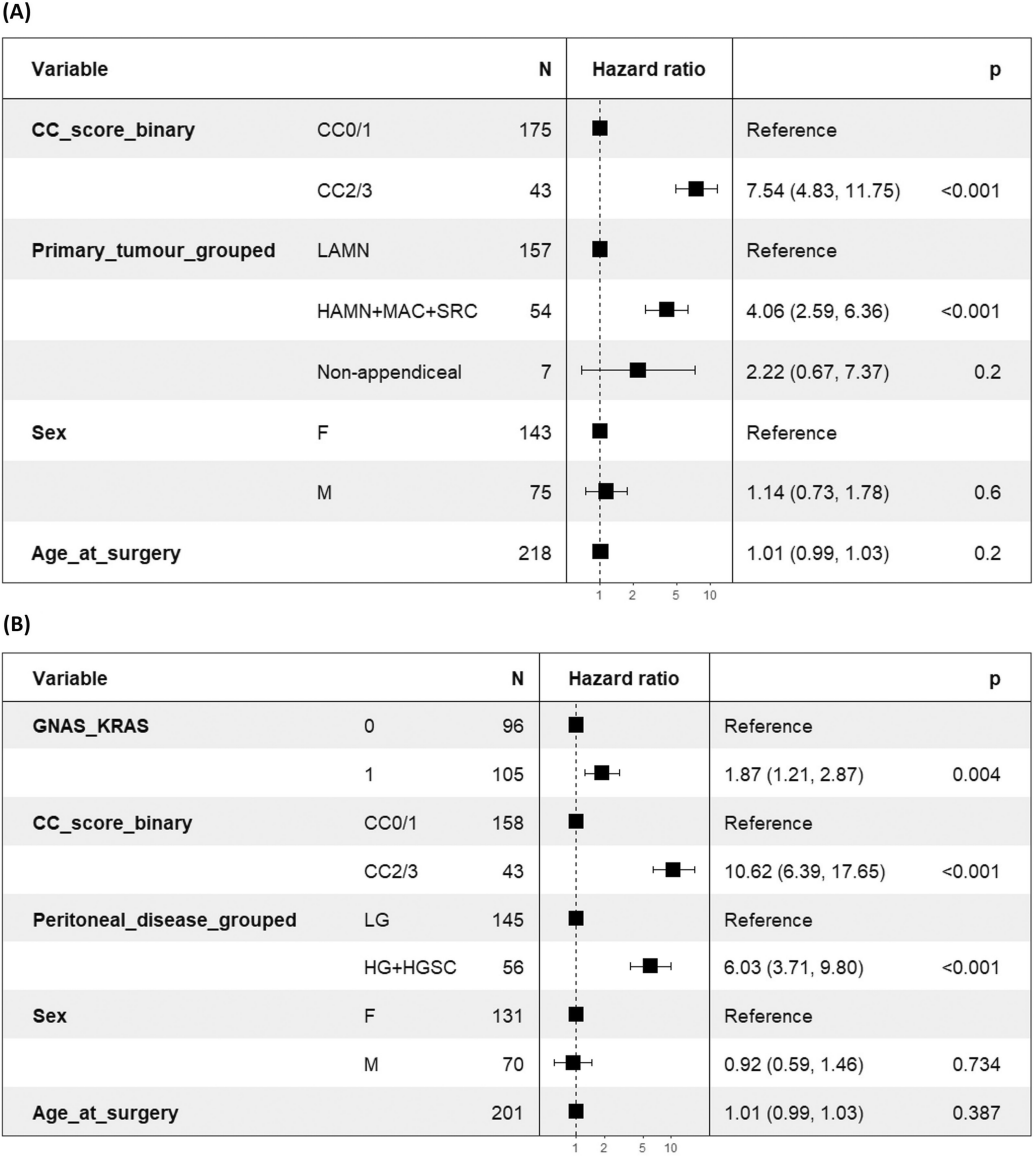
Cox proportional hazards multivariable model forest plot(s) (A) including CC score and primary tumour and (B) peritoneal disease type with acellular mucin (AM) excluded.

As mutations in *GNAS* and *KRAS* genes frequently co‐occur in the same patients, we merged these to create a subset of patients with *KRAS* and/or *GNAS* mutations. This subset had significantly poorer survival than those without mutations at either gene (*p* = 0.001) (Figure S3). When modelling carriage of *KRAS* and/or *GNAS* mutational status (excluding cases with acellular peritoneal mucin), genetic variation in these genes significantly contributed to hazard (*p* = 0.004, HR = 1.87) compared to cases with no mutation in either gene after accounting for CC scoring and peritoneal disease grade (Figure 4B). However, our data indicate that they represent a lesser hazard than peritoneal disease grade.

## 4 Discussion

As expected, the most common mutations were found in *KRAS* and *GNAS*, although at lower frequencies than in some other studies (Figure 1) [20, 21]. Other mutations in our series have generally been reported previously, although unexpected findings were mutated *BAP1* in 2% of patients and mutated *ERBB4* in 3%. *BAP1* is commonly mutated in other neoplasms, such as malignant mesothelioma and melanoma [46, 47]. *ERBB4*, which encodes the tyrosine kinase receptor HER4, has varied effects on cancer development, but mutations in this gene have been implicated in several neoplasms, including melanoma, lung carcinoma and stomach carcinoma [48]. Of the 4 cases with *BAP1* mutation, 3 showed no variants in either *KRAS* or *GNAS*, and of the 7 cases with *ERBB4* mutation, 4 (all LAMNs) likewise showed no variants in either *KRAS* or *GNAS* (Figure 1A). These findings raise the possibility of novel genetic pathways in PMP that may not involve *KRAS* or *GNAS*. Of note in this respect, the targeted hotspot panel was not sufficient to analyse amplifications. Another limitation of the study was a lack of matched normal tissue, which is advantageous in confirming somatic mutation status.

A previous study of mucinous appendiceal neoplasms found wild‐type *GNAS* in 5/6 high‐grade tumours, raising the possibility that high‐grade lesions may not arise from low‐grade tumours [20]. We found *GNAS* mutations in 18/54 (33%) of high‐grade lesions and 73/162 (45%) of low‐grade lesions (Figure 1A); this difference was not significant in our data (*p* = 0.18). For patients with PMP, a potential implication of wild‐type *KRAS* is the possible use of drugs such as cetuximab in treatment. A retrospective study of patients with appendiceal adenocarcinoma showed no survival benefit from cetuximab or panitumumab [49]. However, in a xenograft mouse model of high‐grade PMP, the *KRAS* ^G12D^ inhibitor MRTX1133 was associated with a marked reduction of tumour growth [50]. It could be hypothesised that sequencing of percutaneous or laparoscopic biopsies might identify patients with PMP who could benefit from cetuximab or panitumumab prior to surgery to improve CC0 rates, and further studies in this area are indicated.

Other candidates for targeted therapy are neoplasms with *BRAF* mutations. In a study of patient‐derived organoid and xenograft mouse models of PMP, the *BRAF* ^V600E^ inhibitor encorafenib reduced cell viability and tumour growth [51]. Although *BRAF* mutation in appendiceal neoplasia is rare, the subset of patients harbouring such a mutation might benefit from this treatment and further research is needed.

The low frequency of *APC* and *BRAF* mutation in our series is in keeping with the findings of others and supports the concept that these genes are mutated rarely in pseudomyxoma peritonei, in contrast to colorectal carcinomas [20, 22, 28, 31, 33]. This emphasises the fact that colorectal and appendiceal neoplasms are genetically distinct.

In the 17 specimens consisting of acellular peritoneal mucin, 6 (35%) were positive for mutated DNA (Figure 1B). PMP with acellular mucin has an excellent prognosis [18]. In some cases, it may be that the mucin does contain neoplastic cells, but at such a low concentration that they are missed by routine histopathological assessment. Alternatively, the mucin may be genuinely acellular because the neoplastic cells do not survive after extrusion from the appendix. The detection of mutated DNA in histologically acellular mucin could be consistent with either scenario. In a study of three patients with acellular peritoneal mucin associated with LAMNs harbouring mutated *KRAS*, the *KRAS* mutation was detected in cell‐free DNA in the mucin from all three patients [17].

There is little consistent evidence in the literature about the prognostic impact of genomic alterations in PMP [19, 21, 24]. Our data showed poorer survival was associated with either *KRAS* mutation (*p* = 0.006, HR = 1.72) or *GNA*S mutation (*p* = 0.048, HR = 1.48). Furthermore, the subset of patients with *KRAS* and/or *GNAS* mutations had significantly worse survival than those without mutations at either of these established genes (*p* = 0.001) (Figure S3). *TP53* variants were associated with a trend towards poorer survival, consistent with the findings of others [28]. However, this was not statistically significant in our data (*p* = 0.061). Nevertheless, *TP53* mutations were more likely to be found in high‐grade lesions: they were identified in 9/54 (17%) of high‐grade neoplasms and 7/162 (4%) of low‐grade neoplasms (*p* = 0.007) (Figure 1A), in keeping with other studies [20, 25, 31].

The associations between the various clinical features and survival in our results were generally as expected in the light of previous studies [11, 35, 36]. The CC score influenced survival more than any other factor in our data, and the appendiceal neoplasms showed better overall survival in the LAMN (G1) subgroup compared with the HAMN/MAC/SRC (G2/G3) subgroup. The non‐appendiceal subgroup demonstrated an intermediate survival in this small set of patients (*n* = 7). The grade of the pseudomyxoma peritonei was also significantly associated with survival, and patients with acellular mucin had the best outcome. Survival was more closely associated with the grade of the peritoneal disease than the grade of the primary tumour in cases of PMP of appendiceal origin (Figure S5). This finding supports the practice of grading the primary and peritoneal disease separately in patients with PMP [15].

Our results demonstrate the importance of *KRAS* and *GNAS* variants in the oncogenesis of PMP and are consistent with the conclusions of others that *TP53* appears to be associated with high grade disease. The relative rarity of mutations in other genes suggests we need to look beyond the genes analysed in panels such as the one used in our study to identify other genes that may be commonly involved. The starting point for many studies of the genetics and pathology of appendiceal mucinous neoplasms and PMP is the assumption that they are similar to colorectal carcinoma, but there are many important clinical, pathological and genetic differences between them [19]. We speculate that widening the range of genes studied by NGS sequencing may not only improve prognostic data but also provide insights into the unique behaviour of these tumours. A possible pointer in this direction is our documentation of novel variants in *BAP1* and *ERBB4*. We have also demonstrated that meaningful genetic information can be obtained from PMP that is histologically acellular. Further work in the genetics of PMP may enlighten the whole field of peritoneal metastatic disease, a frequent and clinically difficult condition in patients with common primary cancers of gastrointestinal origin.

## Author Contributions

**Jane Gibson:** formal analysis (equal), writing – review and editing (equal). **Norman John Carr:** conceptualization (equal), data curation (equal), funding acquisition (equal), resources (equal), writing – original draft (equal), writing – review and editing (equal). **Alex Mirnezami:** conceptualization (equal), data curation (equal), funding acquisition (equal), resources (equal), writing – review and editing (equal). **Brendan John Moran:** data curation (equal), resources (equal), writing – review and editing (equal). **Sarah Ennis:** conceptualization (equal), funding acquisition (equal), supervision (equal), writing – review and editing (equal). **Alexios Tzivanakis:** data curation (equal), resources (equal), writing – review and editing (equal). **Konstantinos Boukas:** investigation (equal), writing – review and editing (equal). **Amatta Mirandari:** formal analysis (equal), writing – review and editing (equal). **Reuben J. Pengelly:** formal analysis (equal), writing – review and editing (equal). **Sophia Stanford:** data curation (equal), project administration (equal), resources (equal), writing – review and editing (equal). **Sanjeev Paul Dayal:** data curation (equal), resources (equal), writing – review and editing (equal). **Faheez Mohamed:** data curation (equal), resources (equal), writing – review and editing (equal). **Thomas Desmond Cecil:** data curation (equal), resources (equal), writing – review and editing (equal).

## Ethics Statement

Ethical approval was provided by the National Research Ethics Service, reference 09/H0504/3.

## Conflicts of Interest

The authors declare no conflicts of interest.

## Supporting information


Data S1.

Figure S1.

## Data Availability

The data that support the findings of this study are available on request from the corresponding author. The data are not publicly available due to privacy or ethical restrictions.
