# Supplementary material for: Targeted Genetic Sequencing Analysis of 223 Cases of Pseudomyxoma Peritonei Treated by Cytoreductive Surgery and Hyperthermic Intraperitoneal Chemotherapy Shows Survival Related to GNAS and KRAS Status
Source: Cancer Med. 2024 Oct 22;13(20):e70340. doi: 10.1002/cam4.70340 (PMC11494485; doi:10.1002/cam4.70340)

**Supplementary Figure 1**. Variant allele frequency of curated mutations.


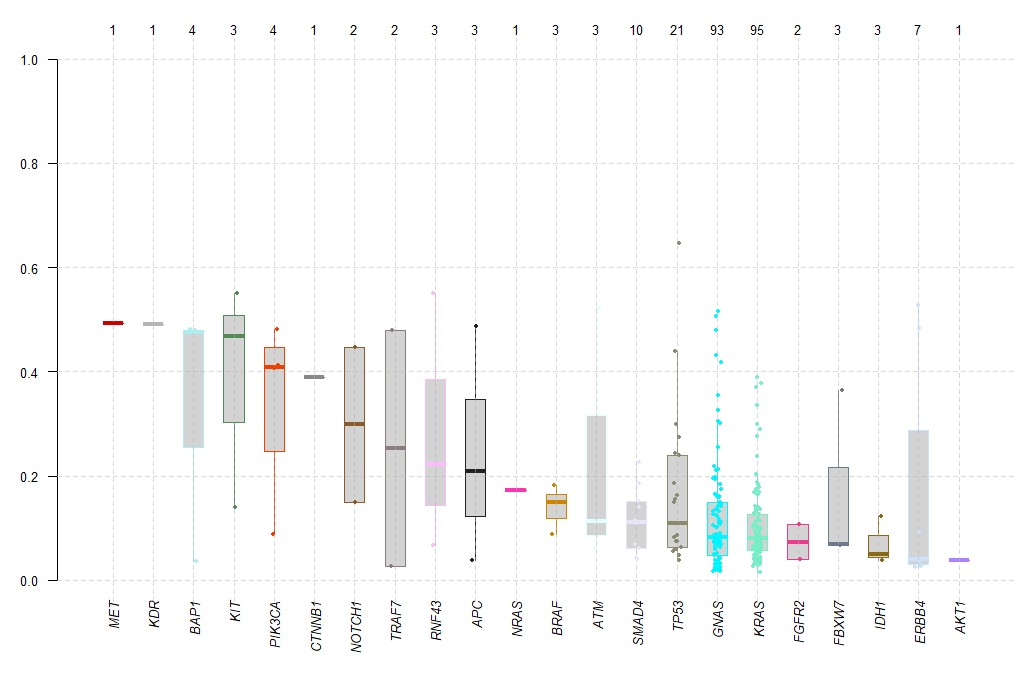


Variant allele frequency

**Supplementary Figure 2.** Lolliplots for mutations present in GNAS and KRAS.


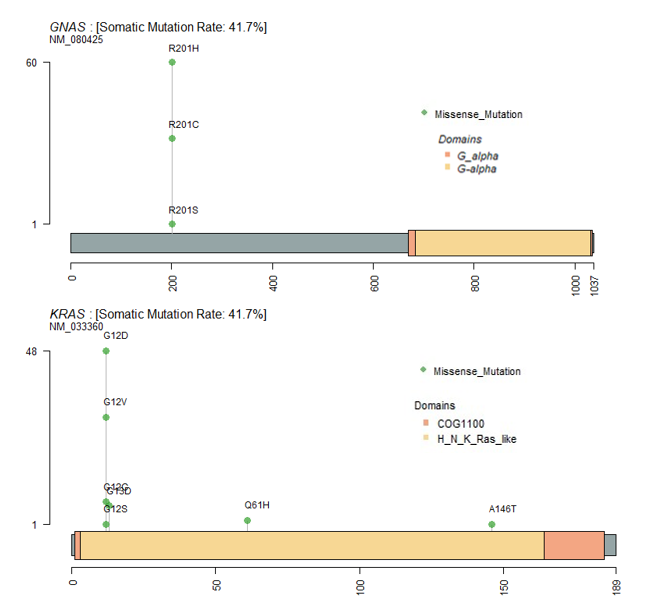


**Supplementary Figure 3.** Kaplan-Meier Survival plot for patients with mutations in KRAS and or GNAS versus the rest.


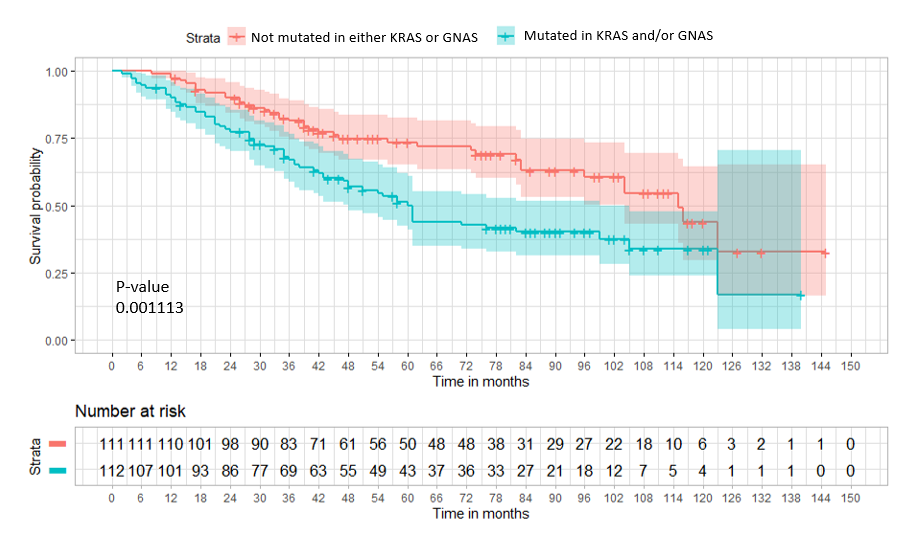


**Supplementary Figure 4.** Cox proportional hazards model forest plot including primary tumour type.


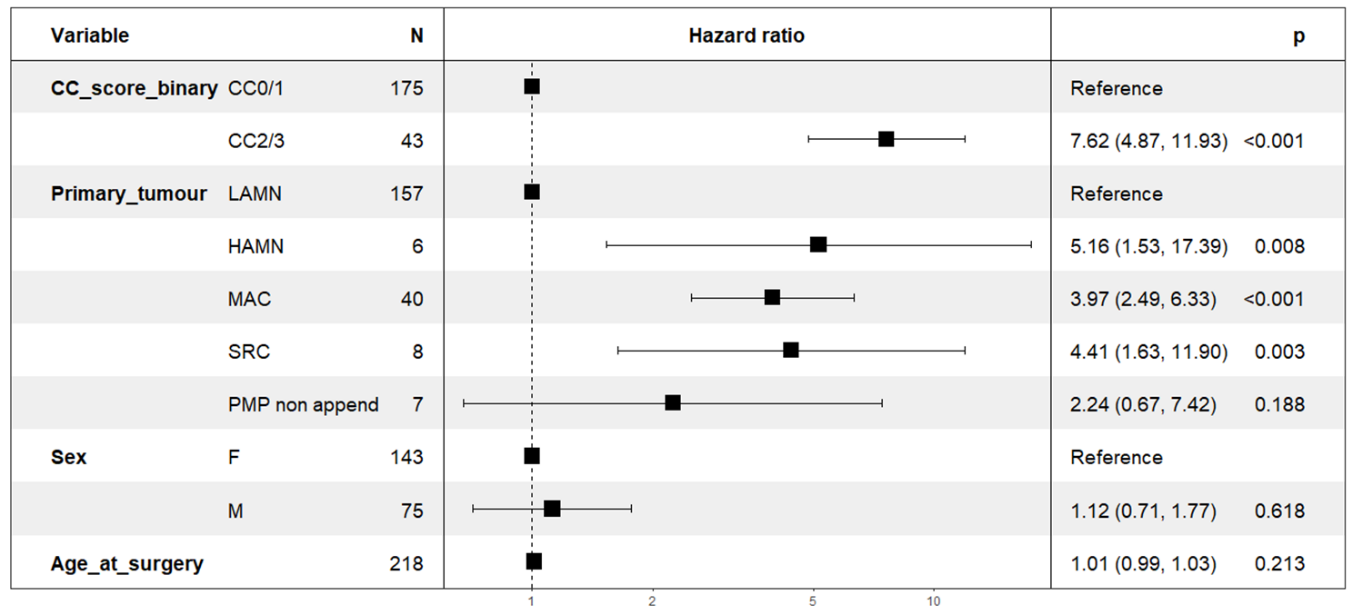


**Supplementary Figure 5.** Cox proportional hazards multivariable model forest plot including primary tumour and peritoneal disease grades. Primary tumour grades codes as 'LAMN'= '1', 'HAMN'= '2', 'MAC' = '2', 'SRC' = ‘3’ (PMP non appendiceal excluded), and peritoneal disease grades as 'LG'= '1', 'HG'= '2', 'HGSC' = 3’ (AM excluded).


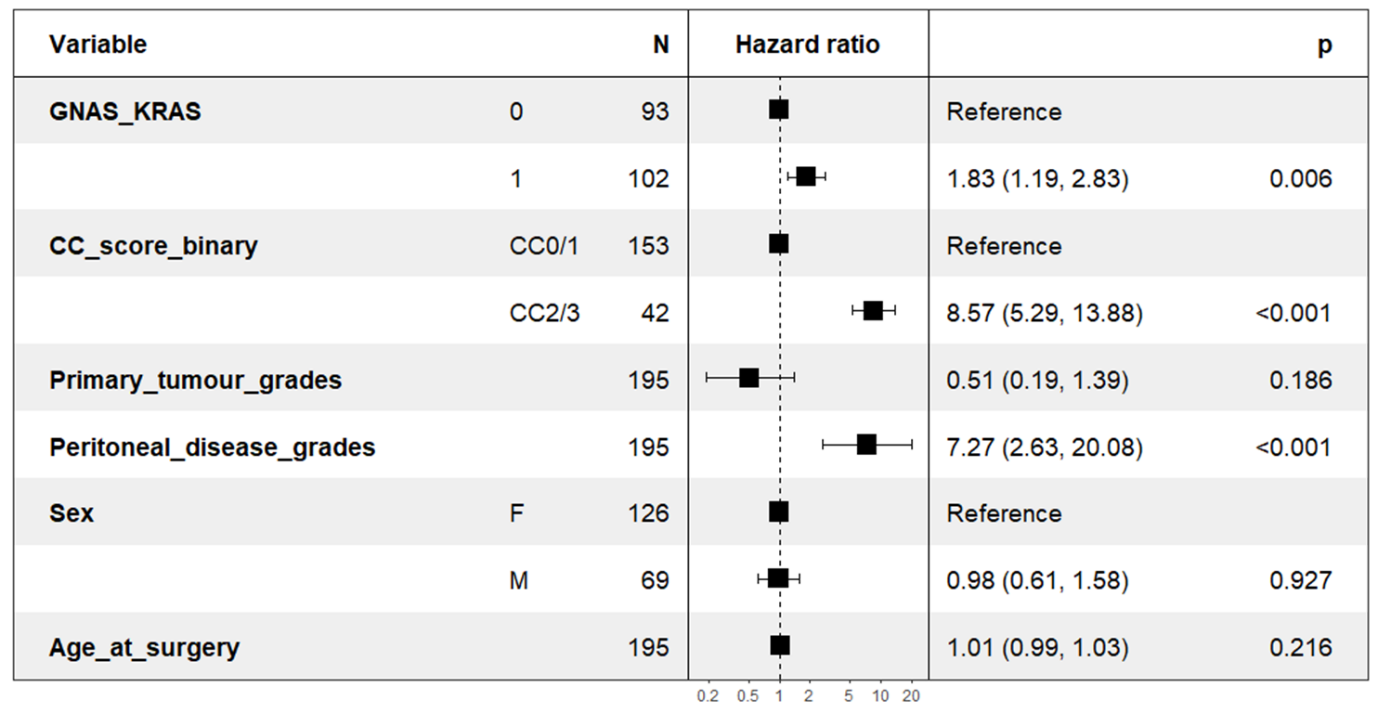

Supplement: Supplementary file 2 — Figure S1. [file CAM4-13-e70340-s001.docx]
